# Integrating plasma proteomics with genome-wide association data to identify novel drug targets for inflammatory bowel disease

**DOI:** 10.1038/s41598-024-66780-w

**Published:** 2024-07-15

**Authors:** Zhongyuan Bai, Jiawei Hao, Miaoran Chen, Kaixin Yao, Leilei Zheng, Liu Liu, Jingxi Hu, Kaiqing Guo, Yongqiang Lv, Feng Li

**Affiliations:** 1 First Clinical Medical School, Shanxi Medical University, Taiyuan, China; 2 Ministry of Education, Key Laboratory of Cellular Physiology at Shanxi Medical University, Taiyuan, China; 3 Hepatobiliary Pancreatogastric Surgery, Shanxi Province Cancer Hospital, Taiyuan, China; 4 Shanxi Hospital Affiliated to Cancer Hospital, Chinese Academy of Medical Sciences, Taiyuan, China; 5 Cancer Hospital Affiliated to Shanxi Medical University, Taiyuan, China; 6 Department of Scientific Research, Shanxi Province Cancer Hospital, Taiyuan, China; 7 Central Laboratory, Shanxi Province Cancer Hospital, Taiyuan, China

**Keywords:** Crohn’s disease, Inflammatory bowel disease, Mendelian randomisation, Proteome-wide association study, Ulcerative colitis, Computational biology and bioinformatics, Genetics, Biomarkers, Gastroenterology, Rheumatology

## Abstract

Inflammatory bowel disease (IBD) is a chronic disease that includes Crohn’s disease (CD) and ulcerative colitis (UC). Although genome-wide association studies (GWASs) have identified many relevant genetic risk loci, the impact of these loci on protein abundance and their potential utility as clinical therapeutic targets remain uncertain. Therefore, this study aimed to investigate the pathogenesis of IBD and identify effective therapeutic targets through a comprehensive and integrated analysis. We systematically integrated GWAS data related to IBD, UC and CD (*N* = 25,305) by the study of de Lange KM with the human blood proteome (*N* = 7213) by the Atherosclerosis Risk in Communities (ARIC) study. Proteome-wide association study (PWAS), mendelian randomisation (MR) and Bayesian colocalisation analysis were used to identify proteins contributing to the risk of IBD. Integrative analysis revealed that genetic variations in IBD, UC and CD affected the abundance of five (*ERAP2, RIPK2, TALDO1, CADM2* and *RHOC*), three (*VSIR, HGFAC and CADM2*) and two (*MST1 and FLRT3*) cis-regulated plasma proteins, respectively (*P* < 0.05). Among the proteins identified via Bayesian colocalisation analysis, *CADM2* was found to be an important common protein between IBD and UC. A drug and five druggable target genes were identified from DGIdb after Bayesian colocalisation analysis. Our study's findings from genetic and proteomic approaches have identified compelling proteins that may serve as important leads for future functional studies and potential drug targets for IBD (UC and CD).

## Introduction

Inflammatory bowel disease (IBD) is a chronic and relapsing gastrointestinal disorder. Although the pathogenesis of IBD remains unclear, it may be driven by a genetic predisposition^1^. IBD primarily includes Crohn’s disease (CD) and ulcerative colitis (UC). The clinical manifestations of CD are more heterogeneous than those of UC. In particular, CD can affect any part of the gastrointestinal tract, especially the terminal ileum or the perianal region. Unlike CD, UC is limited to the large intestine, especially the peri-appendiceal region^2,3^. Despite substantial advancement in the treatment of IBD in the past few years, the standard treatment protocols available at present have limited therapeutic efficacy, with disadvantages such as inadequate response to drugs and development of drug resistance or failure over time. Therefore, it is necessary to identify therapeutic targets to facilitate the development of new drugs.

During the past decade, large-scale genome-wide association studies (GWASs) have identified various non-overlapping genetic risk loci to screen for candidate target genes for drug development. However, the identified variants explain only a minority of the genetic risk^4^. Developing these genetic risk loci into target genes of drugs remains a significant challenge. Katrina et al. identified 25 novel loci through a meta-analysis of GWAS data including 25,305 individuals and provided further insights into the possible mechanisms underlying known therapeutic strategies^5^. However, GWASs have rarely identified causal genes mediating the effects of variation on traits^6^. Therefore, Virginia et al. identified 39 novel susceptibility genes that influence the pathogenesis of IBD by correlating gene expression with characteristics and disease using a new statistical method for a transcriptome-wide association study (TWAS)^7^. Although previous studies have revealed genetic loci associated with the pathogenesis of IBD, their results were based on genomic or transcriptomic (messenger RNA [mRNA]) analyses instead of proteomic analysis. Proteins are the ultimate products of gene expression and may act as potential drug targets and biomarkers^8^.

PWASs have attracted substantial interest in recent years. In PWASs, signals from all variants that collectively affect a protein-coding gene are integrated, and machine learning and predictive models are used to assess their overall implication in protein function^9^. Zhang et al. combined gene and protein expression data to propose the first PWAS framework based on the plasma proteome and identified three specific genetic variants, thereby revealing potential target proteins for the treatment of disease^10^. Altogether, PWAS is a comprehensive, validated and reliable analytical approach.

In this study, we integrated high-throughput proteomic and genetic data derived from plasma samples to identify proteins associated with IBD susceptibility and potential therapeutic targets for IBD. A PWAS was performed to investigate the GWAS and protein quantitative trait locus (pQTL) data of IBD. Independent Mendelian randomisation (MR) was performed to verify the causal relationship between the identified proteins and IBD. Finally, Bayesian co-localisation analysis was performed to examine whether GWAS and pQTL data were consistent with shared causal variants and identified drug and druggable target genes through DGIdb. To the best of our knowledge, this PWAS is the first to report on proteins associated with IBD susceptibility, providing new insights into potential therapeutic approaches for the disease.

## Results

### Associated plasma proteins identified in PWAS

**Figure 1:**
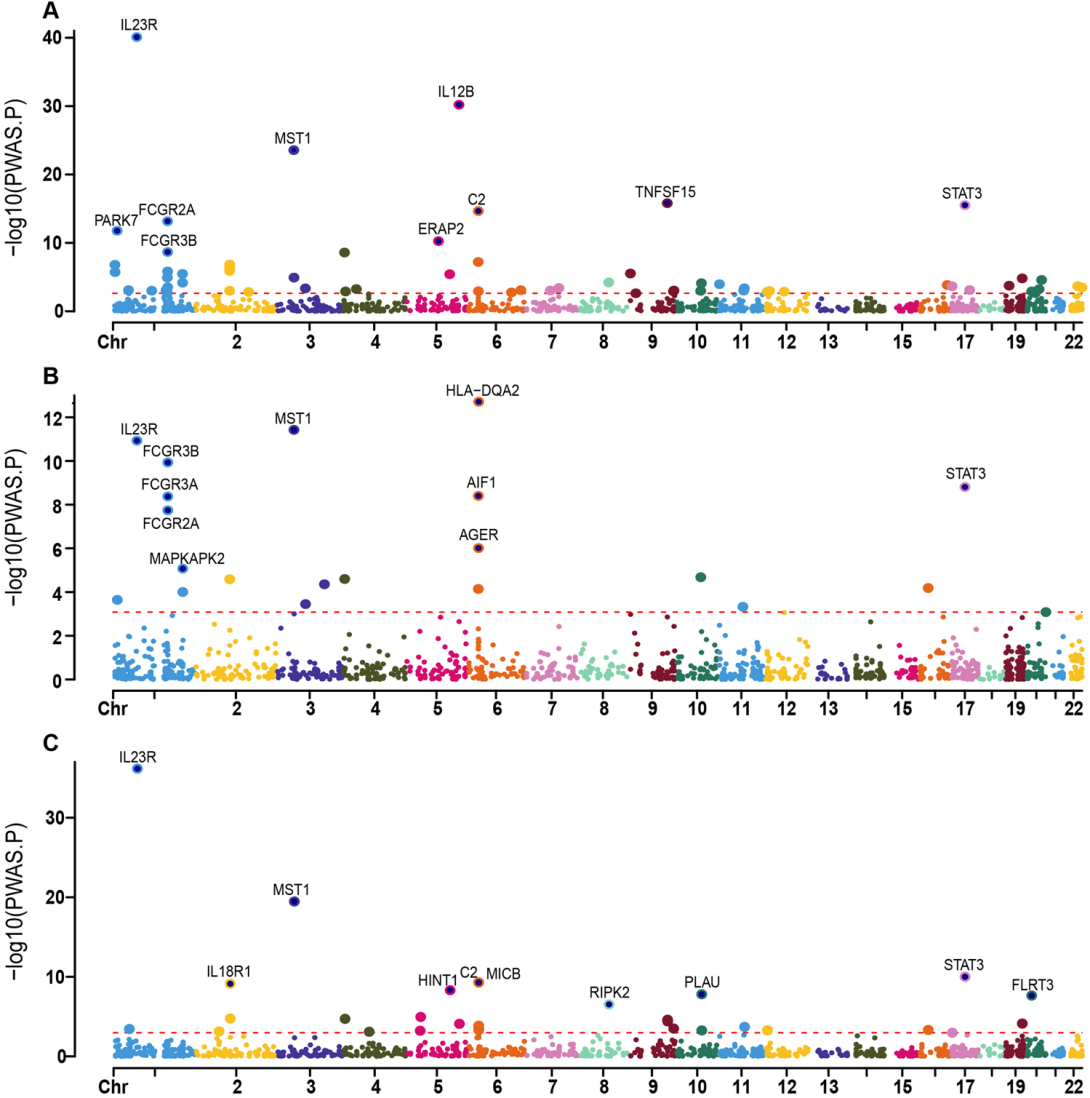
PWAS of IBD (**A**), UC (**B**) and CD (**C**) with the plasma proteomes (*N* = 1348) and GWAS were integrated in Manhattan plot using FUSION Each point in the plot indicates a single association test between a plasma protein and IBD, UC and CD as the -log10 (*P*) of a z-score test result which ordered by genomic position on the *x* axis and the association strength on the *y* axis. 62, 21 and 30 proteins were identified whose cis-regulated plasma protein abundance correlated with IBD, UC and CD, respectively, and the top 10 proteins with the highest correlation are illustrated in the figure. The red horizontal line represents the significant threshold for Bonferroni correction of the FDR *P* < 0.05 which was set at the highest unadjusted P value that is below that in IBD, UC and CD, seperately.

We conducted a PWAS by integrating GWAS data of IBD, UC and CD with the data of 1,348 plasma proteomes using the FUSION pipeline. The abundance of 62, 21 and 30 cis-regulated plasma proteins was significantly associated with IBD, UC and CD, respectively. Of these proteins, 4 were common among IBD, UC and CD. In addition, 15, 17 and 6 proteins were common between IBD and UC, between IBD and CD and between UC and CD, respectively. Each of these proteins had an FDR of < 0.05 with a higher confidence level. Detailed information on plasma proteins associated with IBD, UC and CD is presented in Fig. 1, Table 1 and Supplementary Table S2.

**Table 1:** Candidate top 10 plasma proteins identified by PWAS analysis for IBD, UC and CD.

ID	CHR	PWAS.Z	PWAS.P	PWAS.FDR
IBD				
IL23R	1	13.38	8.10E-41	1.07E-37
IL12B	5	11.56	6.27E-31	4.16E-28
MST1	3	− 10.17	2.75E-24	1.22E-21
TNFSF15	9	− 8.25	1.56E-16	5.17E-14
STAT3	17	8.17	3.06E-16	8.12E-14
C2	6	− 7.93	2.19E-15	4.84E-13
FCGR2A	1	− 7.49	6.78E-14	1.28E-11
PARK7	1	− 7.06	1.69E-12	2.80E-10
ERAP2	5	6.56	5.49E-11	8.09E-09
FCGR3B	1	5.98	2.17E-09	2.88E-07
UC				
HLA-DQA2	6	7.38	1.58E-13	2.10E-10
MST1	3	− 6.97	3.07E-12	2.04E-09
IL23R	1	6.81	9.61E-12	4.25E-09
FCGR3B	1	6.47	9.52E-11	3.16E-08
STAT3	17	− 6.07	1.27E-09	3.37E-07
FCGR3A	1	5.91	3.45E-09	6.54E-07
AIF1	6	− 5.92	3.23E-09	6.54E-07
FCGR2A	1	5.66	1.47E-08	2.44E-06
AGER	6	4.94	7.93E-07	1.17E-04
MAPKAPK2	1	4.50	6.89E-06	9.14E-04
CD				
IL23R	1	12.69	6.84E-37	9.07E-34
MST1	3	− 9.21	3.41E-20	2.26E-17
STAT3	17	− 6.47	1.01E-10	4.46E-08
C2	6	− 6.21	5.39E-10	1.43E-07
MICB	6	6.22	5.05E-10	1.43E-07
IL18R1	2	− 6.15	7.57E-10	1.67E-07
HINT1	5	− 5.85	4.89E-09	9.26E-07
PLAU	10	5.65	1.59E-08	2.64E-06
FLRT3	20	− 5.59	2.32E-08	3.42E-06
RIPK2	8	− 5.12	2.99E-07	3.96E-05

PWAS, proteome-wide association study; ID: ID for the pQTL strongly associated with IBD/UC/CD; PWAS *p*-value: *p*-value for the PWAS association.

### Association of plasma proteins with IBD, UC and CD verified via MR

**Figure 2:**
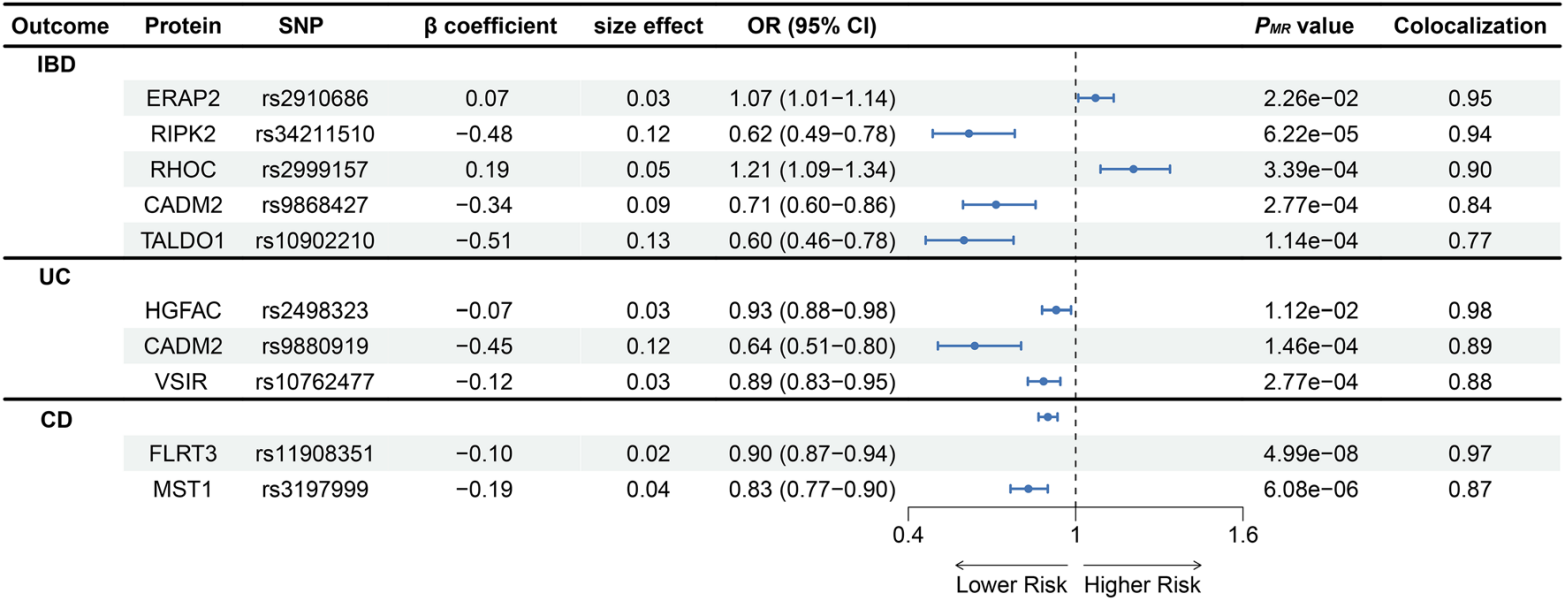
Association of protein expression in the blood with IBD (**A**), UC (**B**) and CD (**C**) risk The forest map for estimates of the relationship between genetically predicted protein levels and IBD, UC and CD.

**Figure 3:**
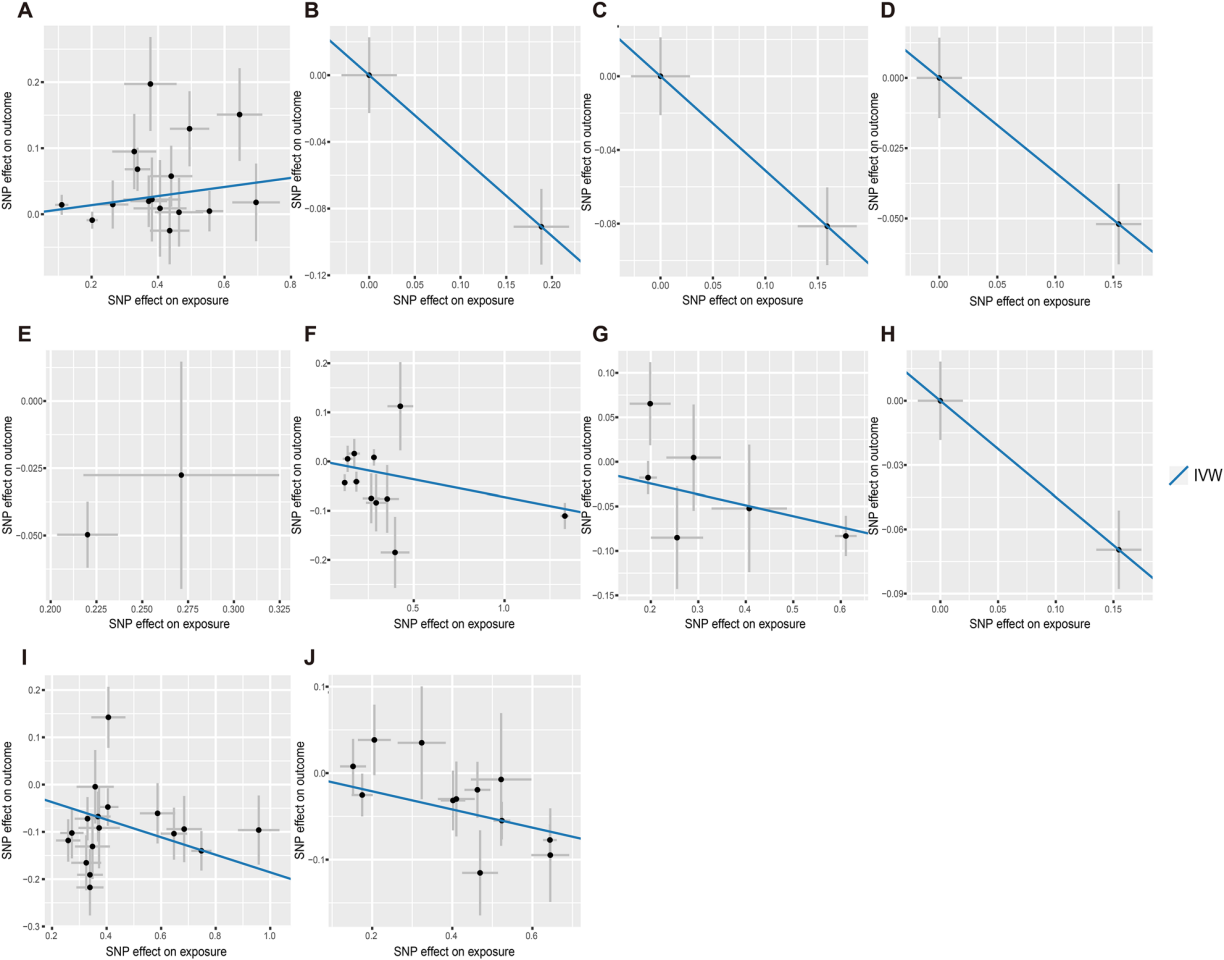
Scatter plots for the MR analysis Scatter plots for IVW highlighting the effect of protein level on IBD, UC and CD. (**A**) ERAP2 (**B**) RIPK2 (**C**) TALDO1 (**D**) CADM2 (**E**) RHOC (**F**) HGFAC (**G**) VSIR (**H**) CADM2 (**I**) MST1 (**J**) FLRT3.

MR was performed to verify the relationship between plasma proteins and the risk of IBD, UC and CD and to elucidate the specific causal relationships. A total of 32, 8 and 9 proteins with strong causal effects were identified as biomarkers for IBD, UC and CD, respectively, (*P* < 0.05). A partial overlap was observed among proteins associated with the risk of IBD, UC and CD. The top five plasma proteins associated with the risk of IBD were MST1 (*P* = 6.14 × 10^−8^, OR = 0.82, 95% CI = 0.77–0.88), PARK7 (*P* = 1.76 × 10^−6^, OR = 0.81, 95% CI = 0.75–0.89), NADK (*P* = 3.25 × 10^−5^, OR = 0.84, 95% CI = 0.78–0.91), RIPK2 (*P* = 6.22 × 10^−5^, OR = 0.62, 95% CI = 0.49–0.78) and TALDO1 (*P* = 1.14 × 10^−4^, OR = 0.60, 95% CI = 0.46–0.78). The top five plasma proteins associated with the risk of UC were MST1 (*P* = 6.22 × 10^−8^, OR = 0.84, 95% CI = 0.79–0.90), CADM2 (*P* = 1.46 × 10^−4^, OR = 0.64, 95% CI = 0.51–0.80), VSIR (*P* = 2.77 × 10^−4^, OR = 0.89, 95% CI = 0.83–0.95), PRKCB (*P* = 6.20 × 10^−4^, OR = 1.19, 95% CI = 1.08–1.31) and PIGR (*P* = 7.14 × 10^−4^, OR = 0.78, 95% CI = 0.67–0.90). The top five plasma proteins associated with the risk of CD were FLRT3 (*P* = 4.99 × 10^−8^, OR = 0.90, 95% CI = 0.87–0.93), MST1 (*P* = 6.08 × 10^−6^, OR = 0.83, 95% CI = 0.77–0.90), ABO (*P* = 3.96 × 10^−5^, OR = 1.11, 95% CI = 1.06–1.16), TNFRSF1A (*P* = 5.32 × 10^−4^, OR = 1.35, 95% CI = 1.14–1.60) and C7 (*P* = 8.41 × 10^−4^, OR = 1.14, 95% CI = 1.06–1.23). Detailed information is provided in Figs 2, 3 and Supplementary Table S1–S5.

In addition to the aforementioned causal proteins, there are potential risk proteins to consider. First, during the process of screening instrumental variables, the thresholds for IL23R, HINT1, and MFNG may have been too stringent, preventing the SNPs of these proteins from being used as instrumental variables to explore the causal relationship between IBD and proteins. The same situation occurs with IL23R, HINT1, RIPK2, and HSPA1A in CD. Fortunately, the PWAS-significant proteins for UC were all utilized as robust instrumental variables for MR analysis. Secondly, under the multiple correction thresholds, some proteins passed the threshold of *P* < 0.05 but did not pass the FDR < 0.05 correction. These include TNFSF15, HGFAC, HYAL1, KLB, HDGF, FCN1, C10orf54, HEBP1, ABO, and MAN2B2 in IBD, IL1R2, and PARK7 in UC, and PPIH, GKN, and SERPINF2 in CD. Additionally, some proteins do not show a significant causal relationship, but their effects on the disease are consistent with the direction of PWAS. These include IL12B, STAT3, FCGR3B, IL1RL1, LRRC32, C2, CD274, CRK, NOG, NCF1, CHRDL2, LY75 and ITLN1 in IBD; STAT3, MICB, HLA-DQA2, IL23R, FCGR3B, FCGR3A, AGER and PCOLCE2 in UC; and C2, TNFSF15, APOM, ADK, IL1RL1, TNFSF8, LRRC32, CFB and C9 in CD.

### Colocalisation of plasma proteins associated with disease risk

**Figure 4:**
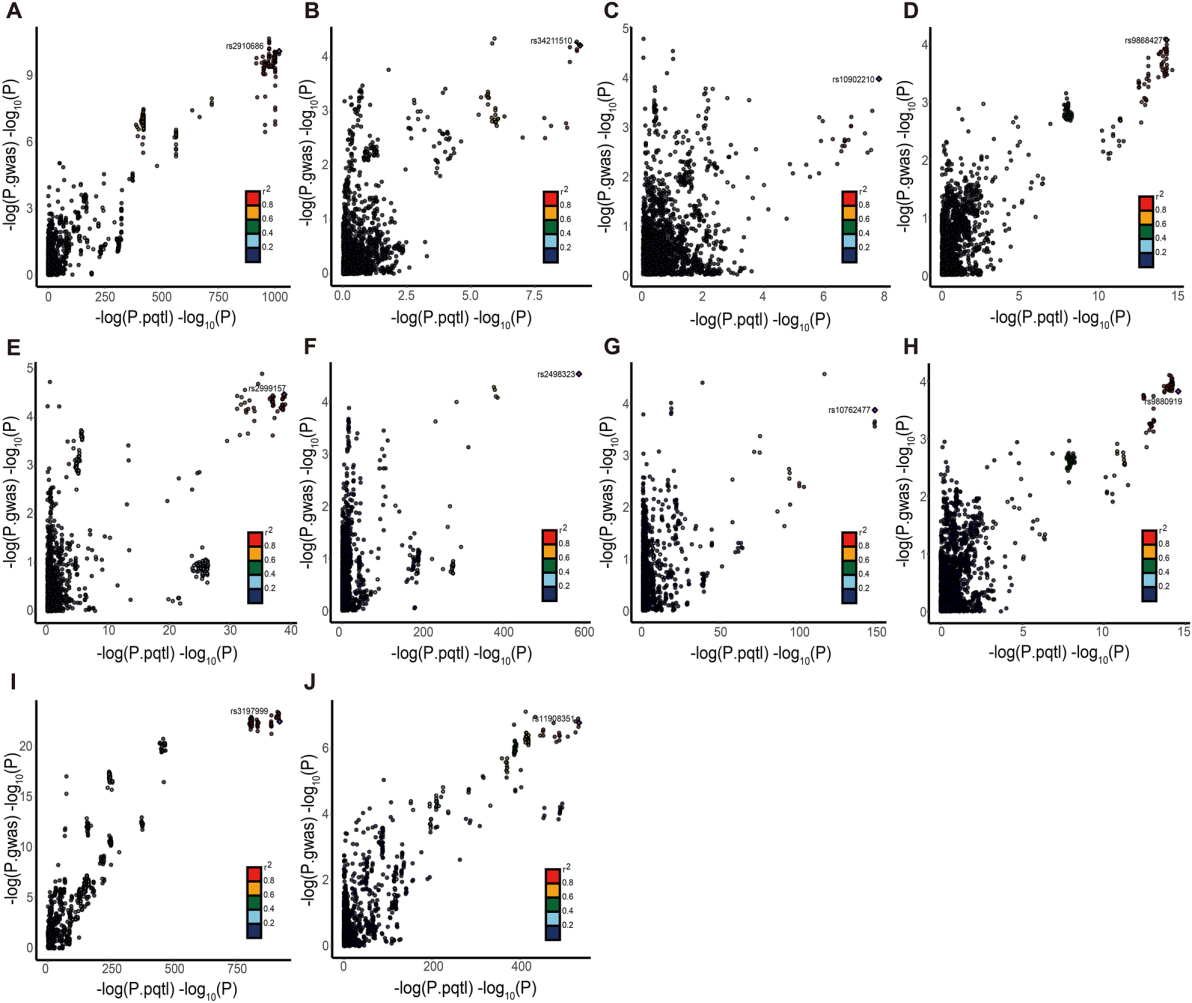
Genetic colocalization of IBD (**A-E**), UC (**F–H**) and CD (**I-J**) (**A**) ERAP2 (**B**) RIPK2 (**C**) TALDO1 (**D**) CADM2 (**E**) RHOC (**F**) HGFAC (**G**) VSIR (**H**) CADM2 (**I**) MST1 (**J**) FLRT3. In this view, each dot is a genetic variant. The SNP with the most notable P value with IBD, UC and CD is marked, and the colors of other SNPs depends on the digit size ordering of linkage disequilibrium (r2). SNPs with missing linkage disequilibrium information are also coded dark blue. In the LocusZoom plots, -log10 (P.gwas) for links with IBD, UC and CD risk are on the *x*-axes, and -log10 (P.pqtl) for relationship with the protein levels on the *y*-axes.

To verify genetic colocalisation, PP was evaluated to identify shared causal variants between pQTL and IBD GWAS data for genes that met the FDR-corrected *P*-value threshold in the MR analysis. The results revealed a probability that the GWAS and pQTL data shared a causal variant (PPH4 ≥ 0.75). Based on the PPH4 value of ≥ 0.75, 5, 3 and 2 proteins were found to play an important role in the progression of IBD, UC and CD (Figs 2 and 4). Among the proteins identified via co-localisation analysis, CADM2 is an important shared protein between IBD and UC. Proteins with PPH3 > 0.7 are also of significant interest. This includes 14 proteins in IBD, 3 in UC, and 3 in CD. Specifically, the proteins in IBD are FCGR2A, PARK7, AIF1, MXRA8, IL1R2, NADK, LY9, PIGR, PLAU, PLCG2, ICAM5, FCGR2B, EPHB4, and AGER. For UC, the proteins are AIF1, PRKCB, and PIGR. In CD, the proteins are C7, IRF3, and TNFRSF1A.

### Drug prediction analysis

As most drugs exert their therapeutic effects through targeting proteins, we finally explored whether the 10 proteins identified through the comprehensive analysis can serve as potential therapeutic targets. Prioritized 4 potential targets for drug therapy intervention, including ERAP2 and RIPK2, CADM2 and VSIR were obtained from the DGIdb, through drug-gene interactions. Through druggability explorations, we identified the inhibitor of ERAP2 Tosedostat as a effective drug of IBD. These findings are expected to promote and facilitate the development of specific drugs for IBD, UC and CD.

## Discussion

In this PWAS, we systematically identified plasma proteins associated with IBD (including UC and CD) through MR and Bayesian analyses to identify potential drug targets. A total of 62, 21 and 30 causal proteins were found to be associated with the risk of IBD, UC and CD, respectively. IBD and UC shared 15 causal proteins, whereas IBD and CD shared 17 causal proteins. Among these proteins, 4 proteins (MST1, IL23R, STAT3 and HGFAC) are common causal proteins associated with the risk of IBD, UC and CD. Co-localisation analysis revealed plasma proteins with higher confidence levels (including 9 plasma proteins); among which, CADM2 may play a crucial role in the pathogenesis of UC and IBD. ERAP2 and RIPK2, CADM2 and VSIR were considered effective plasma proteins associated with IBD.

In recent years, GWASs on IBD have identified more than 240 genetic loci associated with disease risk. The first cross-ethnic association study on IBD by Liu et al. revealed 38 new disease-associated loci based on integrated analysis of genome-wide or immunobrick genotype data from 96,486 individuals. These loci are primarily involved in innate immunity, T-cell signalling and epithelial barrier function^11^. However, given the limited relationship between GWAS-based interpretation of important genes and the pathogenesis of IBD, it is necessary to understand the genetic factors associated with IBD. TWASs link gene expression with genetic susceptibility to disease. In a previous TWAS, more than 250 candidate susceptibility genes were identified in all tissue/cell types, mostly specific to the colonic subsites (ascending, transverse and descending colon)^7^.

Although the abovementioned GWASs and TWASs have been conducted for almost a decade and the relevant research results have been gradually translated into clinical practice, the pharmacological treatment of IBD (UC and CD) remains unsatisfactory, and heterogeneity in the clinical manifestations of the disease makes it difficult to find the best treatment option suitable for all patients^12^. Plasma proteins have superior potential as diagnostic/prognostic biomarkers and therapeutic targets, as they play a critical role in human health and disease^13^. Therefore, understanding the genetic regulation of the proteome may help to identify causal mechanisms underlying complex traits.

Cell adhesion molecule (CADM), a member of the immunoglobulin superfamily and an IBD-associated plasma protein identified in this study, is a pivotal genetic locus associated with various metabolic traits such as obesity and alcohol use^14,15^. Yan et al. reported that deletion of Cadm2 in mice with obesity (Cadm2/ob) reduced adiposity and systemic glucose levels, suggesting a relationship between the expression of CADM and weight gain^16^. Furthermore, a cross-sectional study reported that approximately 15–40% of adults with IBD had obesity, and an additional 20–40% of adults with IBD were overweight^17^. Jostins et al. performed weighted gene co-expression network analysis of loci associated with IBD susceptibility and found that the most significantly enriched module contained 523 genes present in the omental adipose tissue of patients with morbid obesity^18^. Many inflammatory and pro-inflammatory factors are associated with the risk of adverse outcomes of obesity and obesity-related diseases. Specifically, free fatty acids (FFAs) can activate Toll-like receptor (TLR) 4 on the plasma membrane of macrophages, resulting in increased nuclear factor kappa B-dependent expression of pro-inflammatory genes, including TNF-α^19^. In addition, TLR4 present on immune cells, such as monocytes and neutrophils, and non-immune cells, such as adipocytes and endothelial cells, can activate systemic and local inflammatory responses by binding to lipopolysaccharide (LPS), resulting in the dysregulation of metabolic homeostasis and damage to the intestinal mucosal barrier^20,21^. The mucosal barrier is a critical defence against external attack, which is essential for effective relief of the symptoms of IBD and UC. Therefore, the findings of this study suggest that CADM2 deficiency increases the risk of IBD and UC.

Endoplasmic reticulum aminopeptidase 2 (ERAP2), another plasma protein associated with the risk of IBD, is involved in antigen processing and presentation of endogenous peptide antigens via major histocompatibility class I (MHC-I) molecules^22^. It has been reported to be associated with IBD in a previous study^18^, which is consistent with the findings of this study. To date, only two aminopeptidases (ERAP1 and ERAP2) have been identified in the lumen of the endoplasmic reticulum (ER)^23^. ERAP trims antigenic peptide precursors and assists in the presentation of MHC-I molecules; however, peptides presented by MHC-I critically affect antigen presentation to T cells and recognition by NK cells. Therefore, ERAP further contributes to the progression of IBD by affecting innate and adaptive immunity^24,25^. Previous GWAS studies have identified multiple IBD-associated loci that are involved in the specific enrichment of multiple immune cells, including NK cells, and functional dysregulation of the immune system^18^. The role of ERAP2 in the pathogenesis of IBD warrants further investigation. In addition, inhibitors of aminopeptidases (Tosedostat) are promising drugs for the treatment of IBD^26^.

The significant molecular roles of other genes associated with IBD, UC and CD include autophagy in dendritic cells (DCs) and the lectin pathway of complement-MBL. DCs in the intestine play a critical role in inducing immune tolerance and maintaining homeostasis^27^. Receptor-interacting serine-threonine kinase 2 (RIPK-2) induces autophagy in DCs through nucleotide-binding oligomerisation domain 2 (NOD2), which is primarily involved in bacterial processing and generation of MHC-II antigen-specific CD4( +) T cells^28^. Therefore, RIPK-2 may act as a protective factor in IBD and alleviate its symptoms^29,30^. Transaldolase 1 (TALDO1) is a rate-limiting enzyme of the pentose phosphate pathway, and its antibodies are not present in autoimmune diseases except multiple sclerosis. Therefore, the deficiency of TALDO1 may be associated with an increased risk of critical illness^31^. Furthermore, Ras homologue family member C (RHOC) is an important plasma protein associated with IBD susceptibility. It plays an essential role in tumour cell motility and metastasis formation^32^. IBD may be associated with immune-mediated. In this study, ERAP2 and RIPK2, CADM2 and VSIR were identified as druggable target genes using DGIdb, providing novel insights into the development of drugs for treating IBD^33^.

However, this study has some limitations. First, gene expression is a highly complex process that is influenced by multiple factors such as the environment; however, proteomic analysis in this study was limited to the pQTL data of patients of European origin, which may have led to some bias in the results for non-European populations. Second, the primary data in this study were obtained from the plasma proteome of the ARIC cohort which relied strongly on imputation-based approaches for genomic data and did not involve other relevant tissue systems. So, there may be unique pQTL which may not have been captured in our study. Moreover, the effects of uncommon and rare variants and complex trans-associations that remain unknown may play a significant role in heritability and should be investigated in future studies with larger sample sizes.

In conclusion, this study revealed nine protein biomarkers that may contribute to the pathogenesis of IBD (UC and CD). The findings of this study may provide a theoretical basis for future studies on genetic and functional mechanisms, which may help to develop novel therapeutic strategies for IBD (UC and CD).

## Methods

### pQTL data

Circulating proteins (cis-pQTL) were identified based on plasma data on 4,657 plasma proteins from 7,213 European American (EA) individuals recruited by the Atherosclerosis Risk in Communities (ARIC)^10^ study. The study computed the weights of imputation models based on individuals from the Phase-3 1000 Genome Project (1000Genome) and used their genotypic data as weight model. A total of 1,348 unique proteins or protein complexes encoded by autosomal genes were identified using the probabilistic estimation of expression residual (PEER) method, and their data were subjected to rank-based inverse normal transformation and quality control. Genome-wide summary-level statistics for all single-SNP cis-pQTL analysis, irrespective of significance level, and data required to perform PWAS, are available from http://nilanjanchatterjeelab.org/pwas.

### GWAS data

The publicly available GWAS data of IBD, including UC and CD, were obtained from a recent study by de Lange KM. The study included 25,305 individuals of European ancestry (12,160 patients with IBD and 13,145 control individuals) from the UK IBD Genetics Consortium (UKIBDGC) and UK10K Consortium^5^. After quality control, the data on 296,203 variants from 4,474 patients with Crohn’s disease; 4,173 patients with ulcerative colitis; 592 patients with unclassified IBD and 9,500 control individuals were eventually included for analysis. Association summary statistics are available from ftp://ftp.sanger.ac.uk/pub/project/humgen/summary_statistics/human/2016-11-07/.

### Quality control of GWAS data

Only the data of individuals of European ancestry were included in this study. The data were subjected to stringent quality control as follows: (1) aligning data to the hgl9 human reference genome; (2) reserving biallelic autosomal SNPs; (3) removing SNPs with duplicated or missing rs ID; (4) filtering SNPs with MAF values of < 0.01.

### Proteome-wide association study

PWAS was performed using the FUSION standard pipeline by integrating proteomic and genetic data (i.e. expression of plasma proteins in pQTL data and SNPs in GWAS data). For proteins with significant heritability (i.e. heritability *P*-value < 0.01), ENet models, which are a type of more precise predictive model, were used to examine the effects of SNPs on protein abundance by evaluating the relationship of each gene with plasma proteins in IBD, UC and CD. After considering only heritable proteins, the strength of association between plasma protein expression and SNPs of protein cis-positions was examined to predict the effects of SNPs on protein abundance. The linkage disequilibrium (LD) was controlled using an LD reference panel^34^, thereby reducing its impact on the estimated test statistics (http://bogdan.bioinformatics.ucla.edu/software/twas/).

Based on the false discovery rate (FDR) threshold of < 5% and mapping window of ± 500 kb, Z-scores were evaluated to measure the genetic covariance between plasma protein expression and GWAS data^35^. Based on the total number of imputation models for significant cis-heritable plasma proteins or protein complexes, a *P*-value of 3.7 × 10^−5^ indicated significant genetic loci.

### MR analysis

MR analysis, together with a series of sensitivity analyses was performed to verify whether IBD, UC and CD PWAS-significant cis-regulated plasma proteins were associated with IBD abundance and determine candidate directional anchor plasma proteins. The MR analysis conforms to the STROBE-MR Statement^36^, mainly involving instrumental variable selection, instrumental variable assessment, MR analysis as well as sensitivity analysis. We identified protein-specific independent cis-pQTLs through linkage disequilibrium (LD) clumping, using an r^2^ threshold of < 0.01 within the 1 Mb cis-region, based on the European LD reference panel from the 1000 Genomes Project. Subsequently, we harmonized the effect alleles of instrumental variables (IVs) in both pQTLs and outcome GWAS data^37,38^. Notably, the appropriateness of IVs is crucial for MR analysis following stringent inclusion criteria the strength of IVs by the F statistic and removing the weak IVs with the F-statistic less than 10. In cases where only one SNP remained post-selection, we employed the Wald ratio to estimate causality between exposure and outcome^35^. For scenarios with multiple IVs, we employed the inverse-variance weighted (IVW) method^39^, supplemented by MR-Egger to address heterogeneity and horizontal pleiotropy. Horizontal pleiotropy was assessed using the MR-Egger test, where a y-intercept above zero and a *P*-value < 0.05 indicated its presence. Heterogeneity among proteins with multiple IVs was evaluated using Cochran’s Q statistic. Multiple testing corrections were applied using a false discovery rate (FDR) threshold of < 0.05 (Benjamini–Hochberg method). The MR analysis was conducted using the ‘TwoSampleMR’ package in R.

### Bayesian colocalisation analysis

COLOC, a Bayesian test for colocalization analysis was performed to assess the probability of the same causal single-nucleotide variation being responsible for contributing to the risk of IBD (including UC and CD) and modulating the plasma protein levels (i.e. pQTL)^40,41^. The default Coloc priors of p1 = 10^−4^, p2 = 10^−4^ and p12 = 10^−5^ were used. p1 is the probability that a given variant is associated with IBD (including UC and CD), p2 is the probability that a given variant is a significant pQTL and p12 is the probability that a given variant is associated with IBD (including UC and CD) and is a pQTL. The Coloc package in R was used for colocalisation analysis of shared SNPs between pQTL and IBD GWAS datasets and to compute Bayes factors to assess the posterior probability (PP) of five mutually exclusive hypotheses: (1) No SNP is associated with either GWAS or pQTL (H0); (2) Only trait 1 (GWAS) has a causal SNP, whereas no SNP is associated with pQTL (H1); (3) Only trait 2 (pQTL) has a causal SNP, whereas no SNP is associated with GWAS (H2); (4) Both traits (GWAS and pQTL) have independent and different causal SNPs (H3); (5) Both traits have a shared causal SNP (H4). We mainly focused on the last hypothesis (H4), whose PP was denoted as PPH4. A PPH4 of ≥ 0.75 was defined as strong evidence of colocalisation.

### Drug-gene interaction analysis

We explored the interaction between genes and drugs in order to further exploration of the potential of gene-edited plasma proteins after Bayesian colocalization analysis for drug treatment of diseases. The drug–gene interaction database (DGIdb, www.dgidb.org) supports advanced browsing, searching and filtering of information on drug-gene interactions from more than 30 trusted sources^42^. Genes encoding risk plasma proteins are pasted into a database to search for existing drugs or compounds and possible of druggable gene.

### Supplementary Information

Supplementary Tables.

## Data Availability

Publicly available GWAS summary statistics were downloaded from the GWAS catalogue (https://www.ebi.ac.uk/gwas/).

## References

[1] Annese V (2020). Genetics and epigenetics of IBD. Pharmacol. Res..

[2] Graham DB, Xavier RJ (2020). Pathway paradigms revealed from the genetics of inflammatory bowel disease. Nature.

[3] Khor B, Gardet A, Xavier RJ (2011). Genetics and pathogenesis of inflammatory bowel disease. Nature.

[4] McGovern DP, Kugathasan S, Cho JH (2015). Genetics of inflammatory bowel diseases. Gastroenterology.

[5] de Lange KM, Moutsianas L, Lee JC (2017). Genome-wide association study implicates immune activation of multiple integrin genes in inflammatory bowel disease. Nat. Genet..

[6] Gallagher MD, Chen-Plotkin AS (2018). The post-GWAS era: From association to function. Am. J. Hum. Genet..

[7] Diez-Obrero V, Moratalla-Navarro F, Ibanez-Sanz G (2022). Transcriptome-wide association study for inflammatory bowel disease reveals novel candidate susceptibility genes in specific colon subsites and tissue categories. J. Crohns. Colitis.

[8] Rolland D, Basrur V, Jeon YK (2017). Functional proteogenomics reveals biomarkers and therapeutic targets in lymphomas. Proc. Natl. Acad. Sci USA..

[9] Brandes N, Linial N, Linial M (2020). PWAS: Proteome-wide association study-linking genes and phenotypes by functional variation in proteins. Genome Biol..

[10] Zhang J, Dutta D, Kottgen A (2022). Plasma proteome analyses in individuals of European and African ancestry identify cis-pQTLs and models for proteome-wide association studies. Nat. Genet..

[11] Liu JZ, van Sommeren S, Huang H (2015). Association analyses identify 38 susceptibility loci for inflammatory bowel disease and highlight shared genetic risk across populations. Nat. Genet..

[12] Di Sabatino A, Lenti MV, Giuffrida P (2015). New insights into immune mechanisms underlying autoimmune diseases of the gastrointestinal tract. Autoimmun. Rev..

[13] Zhang C, Qin F, Li X (2022). Identification of novel proteins for lacunar stroke by integrating genome-wide association data and human brain proteomes. BMC Med..

[14] Speliotes EK, Willer CJ, Berndt SI (2010). Association analyses of 249,796 individuals reveal 18 new loci associated with body mass index. Nat. Genet..

[15] Pasman JA, Verweij K, Gerring Z (2018). GWAS of lifetime cannabis use reveals new risk loci, genetic overlap with psychiatric traits, and a causal influence of schizophrenia. Nat. Neurosci..

[16] Yan X, Wang Z, Schmidt V (2018). Cadm2 regulates body weight and energy homeostasis in mice. Mol. Metab..

[17] Singh S, Dulai PS, Zarrinpar A (2017). Obesity in IBD: epidemiology, pathogenesis, disease course and treatment outcomes. Nat. Rev. Gastroenterol. Hepatol..

[18] Jostins L, Ripke S, Weersma RK (2012). Host-microbe interactions have shaped the genetic architecture of inflammatory bowel disease. Nature.

[19] Lee JY, Sohn KH, Rhee SH (2001). Saturated fatty acids, but not unsaturated fatty acids, induce the expression of cyclooxygenase-2 mediated through Toll-like receptor 4. J. Biol. Chem..

[20] Manco M, Putignani L, Bottazzo GF (2010). Gut microbiota, lipopolysaccharides, and innate immunity in the pathogenesis of obesity and cardiovascular risk. Endocr. Rev..

[21] Velloso LA, Folli F, Saad MJ (2015). TLR4 at the crossroads of nutrients, gut microbiota, and metabolic inflammation. Endocr. Rev..

[22] Tanioka T, Hattori A, Mizutani S (2005). Regulation of the human leukocyte-derived arginine aminopeptidase/endoplasmic reticulum-aminopeptidase 2 gene by interferon-gamma. FEBS J..

[23] D’Amico S, Tempora P, Lucarini V, Melaiu O, Gaspari S, Algeri M, Fruci D (2021). ERAP1 and ERAP2 Enzymes: A protective shield for RAS against COVID-19?. Int. J. Mol. Sci..

[24] Evnouchidou I, Birtley J, Seregin S (2012). A common single nucleotide polymorphism in endoplasmic reticulum aminopeptidase 2 induces a specificity switch that leads to altered antigen processing. J. Immunol..

[25] Garboczi DN, Ghosh P, Utz U (1996). Structure of the complex between human T-cell receptor, viral peptide and HLA-A2. Nature.

[26] DiNardo CD, Cortes JE (2014). Tosedostat for the treatment of relapsed and refractory acute myeloid leukemia. Expert Opin. Investig. Drugs.

[27] Stagg AJ (2018). Intestinal dendritic cells in health and gut inflammation. Front Immunol..

[28] Larabi A, Barnich N, Nguyen H (2020). New insights into the interplay between autophagy, gut microbiota and inflammatory responses in IBD. Autophagy.

[29] Cooney R, Baker J, Brain O (2010). NOD2 stimulation induces autophagy in dendritic cells influencing bacterial handling and antigen presentation. Nat. Med..

[30] Honjo H, Watanabe T, Kamata K (2021). RIPK2 as a new therapeutic target in inflammatory bowel diseases. Front Pharmacol..

[31] Banki K, Colombo E, Sia F (1994). Oligodendrocyte-specific expression and autoantigenicity of transaldolase in multiple sclerosis. J. Exp. Med..

[32] Wrighton KH (2011). Cytoskeleton: RhoC invades cofilin's space. Nat. Rev. Mol. Cell Biol..

[33] Wagner AH, Coffman AC, Ainscough BJ (2016). DGIdb 2.0: mining clinically relevant drug-gene interactions. Nucl. Acids Res..

[34] Gusev A, Ko A, Shi H (2016). Integrative approaches for large-scale transcriptome-wide association studies. Nat. Genet..

[35] Teumer A (2018). Common methods for performing mendelian randomization. Front Cardiovasc. Med..

[36] Skrivankova VW, Richmond RC, Woolf B (2021). Strengthening the reporting of observational studies in epidemiology using mendelian randomization: The STROBE-MR statement. JAMA.

[37] Davey SG, Hemani G (2014). Mendelian randomization: genetic anchors for causal inference in epidemiological studies. Hum. Mol. Genet..

[38] Tin A, Kottgen A (2021). Mendelian randomization analysis as a tool to gain insights into causes of diseases: A primer. J. Am. Soc. Nephrol..

[39] Kibinge NK, Relton CL, Gaunt TR (2020). Characterizing the causal pathway for genetic variants associated with neurological phenotypes using human brain-derived proteome data. Am. J. Hum. Genet..

[40] Zuber V, Grinberg NF, Gill D (2022). Combining evidence from Mendelian randomization and colocalization: Review and comparison of approaches. Am. J. Hum. Genet..

[41] Giambartolomei C, Vukcevic D, Schadt EE (2014). Bayesian test for colocalisation between pairs of genetic association studies using summary statistics. PLoS Genet.

[42] Freshour SL, Kiwala S, Cotto KC (2021). Integration of the drug-gene interaction database (DGIdb 4.0) with open crowdsource efforts. Nucl. Acids Res..

