# Supplementary material for: Integrating plasma proteomics with genome-wide association data to identify novel drug targets for inflammatory bowel disease
Source: Sci Rep. 2024 Jul 15;14:16251. doi: 10.1038/s41598-024-66780-w (PMC11250821; doi:10.1038/s41598-024-66780-w)
Supplement: Supplementary file 1 — Supplementary Tables. [file 41598_2024_66780_MOESM1_ESM.docx]

**Supplementary Table S1. STROBE-MR checklist of recommended items to address in reports of Mendelian randomization studies**^1^ ^2^

| **Item No.** | **Section** | **Checklist item** | **Relevant text from manuscript** |
| --- | --- | --- | --- |
| 1 | **TITLE and ABSTRACT** | Indicate Mendelian randomization (MR) as the study’s design in the title and/or the abstract if that is a main purpose of the study | Integrating plasma proteomics with genome-wide association data to identify novel drug targets for inflammatory bowel disease |
|  | **INTRODUCTION** |  |  |
| 2 | **Background** | Explain the scientific background and rationale for the reported study. What is the exposure? Is a potential causal relationship between exposure and outcome plausible? Justify why MR is a helpful method to address the study question | Specifically, PWAS as a novel framework can combine gene and protein expression data with the results of GWAS and detect the protein-coding genes associated with phenotypes(eg. IBD, UC and CD) through protein function alterations. However, the effectiveness of plasma protein-coding gene associations with IBD (UC and CD) can only be confirmed by observational studies, which are susceptible to confounding and reverse causation. Moreover, randomized control trials are impractical for exploring the causal associations of thousands of proteins with IBD without conclusive evidence. MR employs genetic variants as instrumental variables for exposures (e.g., proteins) to strengthen causal inference, minimizing confounding and reverse causation. Compared to observational studies, MR optimizes the use of experimental resources and time, avoids redundancy, and enhances the reliable assessment of causal relationships. |
| 3 | **Objectives** | State specific objectives clearly, including pre-specified causal hypotheses (if any). State that MR is a method that, under specific assumptions, intends to estimate causal effects | We performed two-sample MR analysis based on index SNPs for proteins to capture the associations between circulating proteins and the risk of IBD and its subtypes. |
|  | **METHODS** |  |  |
| 4 | **Study design and data sources** | Present key elements of the study design early in the article. Consider including a table listing sources of data for all phases of the study. For each data source contributing to the analysis, describe the following: |  |
|  | a) | Setting: Describe the study design and the underlying population, if possible. Describe the setting, locations, and relevant dates, including periods of recruitment, exposure, follow-up, and data collection, when available. | The publicly available GWAS data of IBD, including UC and CD, were obtained from a recent study by de Lange KM. The study included 25,305 individuals of European ancestry (12,160 patients with IBD and 13,145 control individuals) from the UK IBD Genetics Consortium (UKIBDGC) and UK10K Consortium.(5) After quality control, the data on 296,203 variants from 4,474 patients with Crohn’s disease; 4,173 patients with ulcerative colitis; 592 patients with unclassified IBD and 9,500 control individuals were eventually included for analysis. Association summary statistics are available from ftp://ftp.sanger.ac.uk/pub/project/humgen/summary_statistics/human/2016-11-07/. |
|  | b) | Participants: Give the eligibility criteria, and the sources and methods of selection of participants. Report the sample size, and whether any power or sample size calculations were carried out prior to the main analysis |  |
|  | c) | Describe measurement, quality control and selection of genetic variants |  |
|  | d) | For each exposure, outcome, and other relevant variables, describe methods of assessment and diagnostic criteria for diseases |  |
|  | e) | Provide details of ethics committee approval and participant informed consent, if relevant |  |
| 5 | **Assumptions** | Explicitly state the three core IV assumptions for the main analysis (relevance, independence and exclusion restriction) as well assumptions for any additional or sensitivity analysis | MR analysis, together with a series of sensitivity analyses was performed to verify whether IBD, UC and CD PWAS-significant cis-regulated plasma proteins were associated with IBD abundance and determine candidate directional anchor plasma proteins. The MR analysis conforms to the STROBE-MR Statement,(36) mainly involving instrumental variable selection, instrumental variable assessment, MR analysis as well as sensitivity analysis. We identified protein-specific independent cis-pQTLs through linkage disequilibrium (LD) clumping, using an r2 threshold of < 0.01 within the 1 Mb cis-region, based on the European LD reference panel from the 1000 Genomes Project. Subsequently, we harmonized the effect alleles of instrumental variables (IVs) in both pQTLs and outcome GWAS data.(37, 38) Notably, the appropriateness of IVs is crucial for MR analysis following stringent inclusion criteria. |
| 6 | **Statistical methods: main analysis** | Describe statistical methods and statistics used |  |
|  | a) | Describe how quantitative variables were handled in the analyses (i.e., scale, units, model) | To mitigate potential pleiotropic effects, we employed Phenoscanner to identify and exclude SNPs associated with the IBD, UC and CD. In cases where only one SNP remained post-selection, we employed the Wald ratio to estimate causality between exposure and outcome.(35) For scenarios with multiple IVs, we employed the inverse-variance weighted (IVW) method(39), supplemented by MR-Egger to address heterogeneity and horizontal pleiotropy. Horizontal pleiotropy was assessed using the MR-Egger test, where a y-intercept above zero and a P-value < 0.05 indicated its presence. Heterogeneity among proteins with multiple IVs was evaluated using Cochran’s Q statistic. Multiple testing corrections were applied using a false discovery rate (FDR) threshold of < 0.05 (Benjamini-Hochberg method). The MR analysis was conducted using the ‘TwoSampleMR’ package in R. |
|  | b) | Describe how genetic variants were handled in the analyses and, if applicable, how their weights were selected |  |
|  | c) | Describe the MR estimator (e.g. two-stage least squares, Wald ratio) and related statistics. Detail the included covariates and, in case of two-sample MR, whether the same covariate set was used for adjustment in the two samples |  |
|  | d) | Explain how missing data were addressed |  |
|  | e) | If applicable, indicate how multiple testing was addressed |  |
| 7 | **Assessment of assumptions** | Describe any methods or prior knowledge used to assess the assumptions or justify their validity | Selection of Instrumental Variables |
| 8 | **Sensitivity analyses and additional analyses** | Describe any sensitivity analyses or additional analyses performed (e.g. comparison of effect estimates from different approaches, independent replication, bias analytic techniques, validation of instruments, simulations) | Additionally, to establish the causal effect of proteins on disease risk, we conducted the MR Steiger directionality test to evaluate potential biases from reverse causation, with a significance threshold of P < 0.05. |
| 9 | **Software and pre-registration** |  | TwoSampleMR |
|  | a) | Name statistical software and package(s), including version and settings used |  |
|  | b) | State whether the study protocol and details were pre-registered (as well as when and where) |  |
|  | **RESULTS** |  |  |
| 10 | **Descriptive data** |  | We identified protein-specific independent cis-pQTLs through linkage disequilibrium (LD) clumping, using an r2 threshold of < 0.01 within the 1 Mb cis-region, based on the European LD reference panel from the 1000 Genomes Project. Subsequently, we harmonized the effect alleles of instrumental variables (IVs) in both pQTLs and outcome GWAS data.(37, 38) Notably, the appropriateness of IVs is crucial for MR analysis following stringent inclusion criteria. |
|  | a) | Report the numbers of individuals at each stage of included studies and reasons for exclusion. Consider use of a flow diagram |  |
|  | b) | Report summary statistics for phenotypic exposure(s), outcome(s), and other relevant variables (e.g. means, SDs, proportions) |  |
|  | c) | If the data sources include meta-analyses of previous studies, provide the assessments of heterogeneity across these studies |  |
|  | d) | For two-sample MR:  i.  Provide justification of the similarity of the genetic variant-exposure associations between the exposure and outcome samples  ii.  Provide information on the number of individuals who overlap between the exposure and outcome studies |  |
| 11 | **Main results** |  | MR was performed to verify the relationship between plasma proteins and the risk of IBD, UC and CD and to elucidate the specific causal relationships. A total of 32, 8 and 9 proteins with strong causal effects were identified as biomarkers for IBD, UC and CD, respectively, (P < 0.05). A partial overlap was observed among proteins associated with the risk of IBD, UC and CD. The top five plasma proteins associated with the risk of IBD were MST1 (P = 6.14 × 10^-8^, OR = 0.82, 95% CI = 0.77–0.88), PARK7 (P = 1.76 × 10^-6^, OR = 0.81, 95% CI = 0.75–0.89), NADK (P = 3.25 × 10^-5^, OR = 0.84, 95% CI = 0.78–0.91), RIPK2 (P = 6.22 × 10^-5^, OR = 0.62, 95% CI = 0.49–0.78) and TALDO1 (P = 1.14 × 10^-4^, OR = 0.60, 95% CI = 0.46–0.78). The top five plasma proteins associated with the risk of UC were MST1 (P = 6.22 × 10^-8^, OR = 0.84, 95% CI = 0.79–0.90), CADM2 (P = 1.46 × 10^-4^, OR = 0.64, 95% CI = 0.51–0.80), VSIR (P = 2.77 × 10^-4^, OR = 0.89, 95% CI = 0.83–0.95), PRKCB (P = 6.20 × 10^-4^, OR = 1.19, 95% CI = 1.08–1.31) and PIGR (P = 7.14×10^-4^, OR = 0.78, 95% CI = 0.67–0.90). The top five plasma proteins associated with the risk of CD were FLRT3 (P = 4.99 × 10^-8^, OR = 0.90, 95% CI = 0.87–0.93), MST1 (P = 6.08 × 10^-6^, OR = 0.83, 95% CI = 0.77–0.90), ABO (P = 3.96 × 10^-5^, OR = 1.11, 95% CI = 1.06–1.16), TNFRSF1A (P = 5.32 × 10^-4^, OR = 1.35, 95% CI = 1.14–1.60) and C7 (P = 8.41 × 10^-4^, OR = 1.14, 95% CI = 1.06–1.23). Detailed information is provided in Figures 2 and 3 and Supplementary Tables 3–5. |
|  | a) | Report the associations between genetic variant and exposure, and between genetic variant and outcome, preferably on an interpretable scale |  |
|  | b) | Report MR estimates of the relationship between exposure and outcome, and the measures of uncertainty from the MR analysis, on an interpretable scale, such as odds ratio or relative risk per SD difference |  |
|  | c) | If relevant, consider translating estimates of relative risk into absolute risk for a meaningful time period |  |
|  | d) | Consider plots to visualize results (e.g. forest plot, scatterplot of associations between genetic variants and outcome versus between genetic variants and exposure) |  |
| 12 | **Assessment of assumptions** |  | For a total of protein-IBD pairs that are significantly reported from the IVW method, we performed MR-Egger analysis to test the pleiotropy as well as calculated Cochran’s Q statistic to test heterogeneity. |
|  | a) | Report the assessment of the validity of the assumptions |  |
|  | b) | Report any additional statistics (e.g., assessments of heterogeneity across genetic variants, such as *I^2^*, Q statistic or E-value) |  |
| 13 | **Sensitivity analyses and additional analyses** |  |  |
|  | a) | Report any sensitivity analyses to assess the robustness of the main results to violations of the assumptions |  |
|  | b) | Report results from other sensitivity analyses or additional analyses | Bayesian co-localization analysisThe correlation among causal proteins and current drug targets for IBD |
|  | c) | Report any assessment of direction of causal relationship (e.g., bidirectional MR) |  |
|  | d) | When relevant, report and compare with estimates from non-MR analyses |  |
|  | e) | Consider additional plots to visualize results (e.g., leave-one-out analyses) |  |
|  | **DISCUSSION** |  |  |
| 14 | **Key results** | Summarize key results with reference to study objectives | In this PWAS, we systematically identified plasma proteins associated with IBD (including UC and CD) through MR and Bayesian analyses to identify potential drug targets. A total of 62, 21 and 30 causal proteins were found to be associated with the risk of IBD, UC and CD, respectively. IBD and UC shared 15 causal proteins, whereas IBD and CD shared 17 causal proteins. Among these proteins, 4 proteins (MST1, IL23R, STAT3 and HGFAC) are common causal proteins associated with the risk of IBD, UC and CD. Co-localisation analysis revealed plasma proteins with higher confidence levels (including 9 plasma proteins); among which, CADM2 may play a crucial role in the pathogenesis of UC and IBD. ERAP2 and RIPK2, CADM2 and VSIR were considered effective plasma proteins associated with IBD. |
| 15 | **Limitations** | Discuss limitations of the study, taking into account the validity of the IV assumptions, other sources of potential bias, and imprecision. Discuss both direction and magnitude of any potential bias and any efforts to address them | However, this study has some limitations. First, gene expression is a highly complex process that is influenced by multiple factors such as the environment; however, proteomic analysis in this study was limited to the pQTL data of patients of European origin, which may have led to some bias in the results for non-European populations. Second, the primary data in this study were obtained from the plasma proteome of the ARIC cohort which relied strongly on imputation-based approaches for genomic data and did not involve other relevant tissue systems. So, there may be unique pQTL which may not have been captured in our study. Moreover, the effects of uncommon and rare variants and complex trans-associations that remain unknown may play a significant role in heritability and should be investigated in future studies with larger sample sizes. |
| 16 | **Interpretation** |  | Discussion |
|  | a) | Meaning: Give a cautious overall interpretation of results in the context of their limitations and in comparison with other studies |  |
|  | b) | Mechanism: Discuss underlying biological mechanisms that could drive a potential causal relationship between the investigated exposure and the outcome, and whether the gene-environment equivalence assumption is reasonable. Use causal language carefully, clarifying that IV estimates may provide causal effects only under certain assumptions |  |
|  | c) | Clinical relevance: Discuss whether the results have clinical or public policy relevance, and to what extent they inform effect sizes of possible interventions |  |
| 17 | **Generalizability** | Discuss the generalizability of the study results (a) to other populations, (b) across other exposure periods/timings, and (c) across other levels of exposure | First, gene expression is a highly complex process that is influenced by multiple factors such as the environment; however, proteomic analysis in this study was limited to the pQTL data of patients of European origin, which may have led to some bias in the results for non-European populations. |
|  | **OTHER INFORMATION** |  |  |
| 18 | **Funding** | Describe sources of funding and the role of funders in the present study and, if applicable, sources of funding for the databases and original study or studies on which the present study is based | This work was supported by grants from the Key Project of Health Commission of Shanxi Province (2022XM28 to Feng Li). |
| 19 | **Data and data sharing** | Provide the data used to perform all analyses or report where and how the data can be accessed, and reference these sources in the article. Provide the statistical code needed to reproduce the results in the article, or report whether the code is publicly accessible and if so, where | Publicly available GWAS summary statistics were downloaded from the GWAS catalogue (https://www.ebi.ac.uk/). |
| 20 | **Conflicts of Interest** | All authors should declare all potential conflicts of interest | The authors declare that the research was conducted in the absence of any commercial or financial relationships that could be construed as a potential conflict of interest. |

This checklist is copyrighted by the Equator Network under the Creative Commons Attribution 3.0 Unported (CC BY 3.0) license.

1. Skrivankova VW, Richmond RC, Woolf BAR, Yarmolinsky J, Davies NM, Swanson SA, et al. Strengthening the Reporting of Observational Studies in Epidemiology using Mendelian Randomization (STROBE-MR) Statement. JAMA. 2021;under review.

2. Skrivankova VW, Richmond RC, Woolf BAR, Davies NM, Swanson SA, VanderWeele TJ, et al. Strengthening the Reporting of Observational Studies in Epidemiology using Mendelian Randomisation (STROBE-MR): Explanation and Elaboration. BMJ. 2021;375:n2233.

**Supplementary Table S2. PWAS-MR-COLOC Results**

| **protein** | **PWAS.Z** | **PWAS.P** | **PWAS.FDR** | **OR** | **(95%CI)** | | **pval** | **F** | **EGGER** | **INT.P** | **PP.H3** | **PP.H4** |
| --- | --- | --- | --- | --- | --- | --- | --- | --- | --- | --- | --- | --- |
| **IBD** |  |  |  |  |  |  |  |  |  |  |  |  |
| IL23R | 13.38 | 8.10E-41 | 1.07E-37 |  |  |  |  |  |  |  |  |  |
| IL12B | 11.56 | 6.27E-31 | 4.16E-28 | 1.13 | 0.97 | 1.31 | 1.20E-01 | 213 | 3.54E-01 | 1.87E-01 |  |  |
| MST1 | -10.17 | 2.75E-24 | 1.22E-21 | 0.82 | 0.77 | 0.88 | 6.14E-08 | 278 | 3.81E-01 | 3.20E-04 | 0.47 | 0.53 |
| TNFSF15 | -8.25 | 1.56E-16 | 5.17E-14 | 0.79 | 0.62 | 1.00 | 4.55E-02 | 45 |  |  |  |  |
| STAT3 | 8.17 | 3.06E-16 | 8.12E-14 | 1.01 | 0.72 | 1.43 | 9.42E-01 | 339 |  |  |  |  |
| C2 | -7.93 | 2.19E-15 | 4.84E-13 | 0.89 | 0.77 | 1.01 | 7.94E-02 | 100 | 9.75E-01 | 5.65E-01 |  |  |
| FCGR2A | -7.49 | 6.78E-14 | 1.28E-11 | 0.90 | 0.83 | 0.96 | 3.33E-03 | 268 | 3.54E-01 | 8.76E-01 | 1 | 0 |
| PARK7 | -7.06 | 1.69E-12 | 2.80E-10 | 0.81 | 0.75 | 0.89 | 1.76E-06 | 194 | 2.19E-01 | 4.03E-01 | 0.93 | 0.07 |
| ERAP2 | 6.56 | 5.49E-11 | 8.09E-09 | 1.07 | 1.01 | 1.14 | 2.26E-02 | 433 | 1.41E-01 | 6.67E-01 | 0.05 | 0.95 |
| FCGR3B | 5.98 | 2.17E-09 | 2.88E-07 | 1.19 | 0.98 | 1.46 | 8.36E-02 | 37 | 4.53E-01 | 7.80E-01 |  |  |
| HGFAC | -5.96 | 2.58E-09 | 3.11E-07 | 0.91 | 0.83 | 1.00 | 4.26E-02 | 440 | 5.20E-01 | 9.72E-01 |  |  |
| AIF1 | -5.41 | 6.16E-08 | 6.81E-06 | 0.79 | 0.70 | 0.89 | 2.02E-04 | 56 | 4.61E-01 | 9.17E-01 | 1 | 0 |
| IL1RL1 | 5.26 | 1.46E-07 | 1.49E-05 | 1.02 | 0.92 | 1.13 | 7.11E-01 | 271 | 2.32E-01 | 1.36E-01 |  |  |
| MXRA8 | -5.24 | 1.60E-07 | 1.52E-05 | 0.72 | 0.59 | 0.88 | 1.24E-03 | 55 |  |  | 0.78 | 0.22 |
| IL18R1 | 5.11 | 3.26E-07 | 2.88E-05 | 1.00 | 0.94 | 1.06 | 9.18E-01 | 465 | 9.13E-01 | 8.42E-01 |  |  |
| IL1R2 | 4.85 | 1.25E-06 | 1.04E-04 | 1.20 | 1.04 | 1.38 | 1.35E-02 | 256 | 2.83E-01 | 4.28E-01 | 1 | 0 |
| FCGR3A | 4.81 | 1.52E-06 | 1.19E-04 | 0.98 | 0.89 | 1.09 | 7.40E-01 | 255 | 2.70E-01 | 2.89E-01 |  |  |
| NADK | -4.76 | 1.89E-06 | 1.39E-04 | 0.84 | 0.78 | 0.91 | 3.25E-05 | 363 |  |  | 0.77 | 0.23 |
| CD274 | -4.67 | 3.01E-06 | 2.10E-04 | 0.96 | 0.76 | 1.22 | 7.43E-01 | 150 |  |  |  |  |
| MAPKAPK2 | 4.64 | 3.43E-06 | 2.27E-04 | 0.84 | 0.67 | 1.06 | 1.43E-01 | 137 |  |  |  |  |
| HINT1 | -4.62 | 3.79E-06 | 2.39E-04 |  |  |  |  |  |  |  |  |  |
| LY9 | 4.38 | 1.20E-05 | 6.92E-04 | 1.09 | 1.01 | 1.18 | 3.07E-02 | 194 | 8.33E-01 | 2.58E-01 | 1 | 0 |
| HYAL1 | -4.38 | 1.20E-05 | 6.92E-04 | 1.49 | 1.03 | 2.14 | 3.40E-02 | 72 |  |  |  |  |
| IRF3 | -4.32 | 1.55E-05 | 8.56E-04 | 0.92 | 0.88 | 0.96 | 2.04E-04 | 181 | 2.06E-01 | 9.61E-01 | 0.31 | 0.34 |
| MMP9 | -4.20 | 2.65E-05 | 1.41E-03 | 0.87 | 0.80 | 0.94 | 9.70E-04 | 156 |  |  | 0.49 | 0.47 |
| RIPK2 | -4.01 | 6.00E-05 | 3.06E-03 | 0.62 | 0.49 | 0.78 | 6.22E-05 | 38 |  |  | 0.02 | 0.94 |
| PIGR | -3.99 | 6.69E-05 | 3.29E-03 | 0.87 | 0.78 | 0.98 | 1.75E-02 | 88 |  |  | 1 | 0 |
| PLAU | -3.95 | 7.86E-05 | 3.72E-03 | 0.88 | 0.81 | 0.97 | 7.50E-03 | 131 | 3.68E-01 | 7.09E-01 | 0.81 | 0.18 |
| TALDO1 | -3.87 | 1.10E-04 | 5.03E-03 | 0.60 | 0.46 | 0.78 | 1.14E-04 | 32 |  |  | 0.09 | 0.77 |
| PLCG2 | 3.80 | 1.44E-04 | 6.36E-03 | 1.12 | 1.03 | 1.21 | 6.45E-03 | 119 | 9.17E-01 | 5.40E-01 | 1 | 0 |
| ICAM5 | 3.74 | 1.81E-04 | 7.74E-03 | 1.06 | 1.03 | 1.10 | 1.02E-03 | 342 | 9.29E-02 | 8.34E-01 | 1 | 0 |
| MFNG | -3.71 | 2.12E-04 | 8.78E-03 |  |  |  |  |  |  |  |  |  |
| CRK | -3.69 | 2.22E-04 | 8.92E-03 | 0.71 | 0.46 | 1.09 | 1.17E-01 | 20 |  |  |  |  |
| FCGR2B | -3.61 | 3.09E-04 | 1.17E-02 | 0.90 | 0.84 | 0.96 | 2.63E-03 | 234 | 3.81E-01 | 4.85E-01 | 1 | 0 |
| TYMP | -3.60 | 3.25E-04 | 1.20E-02 | 0.89 | 0.83 | 0.96 | 2.33E-03 | 138 | 4.41E-01 | 9.75E-01 | 0.08 | 0.17 |
| EPHB4 | 3.55 | 3.92E-04 | 1.40E-02 | 1.20 | 1.09 | 1.32 | 2.24E-04 | 248 |  |  | 0.9 | 0.08 |
| LRRC32 | 3.52 | 4.36E-04 | 1.52E-02 | 1.03 | 0.84 | 1.26 | 7.75E-01 | 26 |  |  |  |  |
| CADM2 | -3.51 | 4.50E-04 | 1.53E-02 | 0.72 | 0.60 | 0.86 | 2.77E-04 | 63 |  |  | 0.05 | 0.84 |
| KLB | 3.45 | 5.68E-04 | 1.88E-02 | 1.05 | 1.01 | 1.08 | 6.36E-03 | 193 | 4.37E-03 | 7.61E-02 |  |  |
| BPI | 3.43 | 5.94E-04 | 1.92E-02 | 1.06 | 1.02 | 1.10 | 2.18E-03 | 308 | 2.51E-01 | 9.16E-01 | 0.12 | 0.16 |
| GUCA2B | 3.35 | 8.07E-04 | 2.47E-02 | 1.25 | 1.07 | 1.46 | 5.85E-03 | 86 |  |  | 0.15 | 0.64 |
| PLG | 3.35 | 8.20E-04 | 2.47E-02 | 1.09 | 1.04 | 1.15 | 4.76E-04 | 149 | 2.04E-01 | 9.72E-01 | 0.07 | 0.07 |
| NOG | -3.36 | 7.92E-04 | 2.47E-02 | 0.89 | 0.76 | 1.02 | 1.00E-01 | 50 |  |  |  |  |
| HDGF | -3.32 | 9.06E-04 | 2.59E-02 | 0.94 | 0.91 | 0.98 | 2.06E-03 | 283 | 1.20E-02 | 4.38E-01 |  |  |
| RHOC | 3.30 | 9.73E-04 | 2.59E-02 | 1.21 | 1.09 | 1.34 | 3.39E-04 | 103 |  |  | 0.06 | 0.9 |
| IL1R1 | -3.30 | 9.77E-04 | 2.59E-02 | 1.00 | 0.90 | 1.12 | 9.71E-01 | 88 |  |  |  |  |
| NCF1 | -3.31 | 9.30E-04 | 2.59E-02 | 0.93 | 0.81 | 1.07 | 2.85E-01 | 104 | 1.66E-01 | 3.05E-02 |  |  |
| FCN1 | 3.31 | 9.33E-04 | 2.59E-02 | 1.08 | 1.04 | 1.13 | 1.57E-04 | 156 | 2.73E-02 | 8.41E-01 |  |  |
| CHRDL2 | -3.30 | 9.53E-04 | 2.59E-02 | 0.92 | 0.80 | 1.05 | 2.26E-01 | 59 |  |  |  |  |
| VSIR | -3.28 | 1.03E-03 | 2.68E-02 | 0.94 | 0.89 | 0.99 | 1.23E-02 | 163 | 4.60E-02 | 3.68E-01 |  |  |
| HEBP1 | 3.26 | 1.10E-03 | 2.81E-02 | 1.05 | 1.00 | 1.09 | 4.06E-02 | 333 | 1.47E-01 | 8.44E-01 |  |  |
| AGER | 3.25 | 1.17E-03 | 2.93E-02 | 1.14 | 1.06 | 1.23 | 6.53E-04 | 200 |  |  | 1 | 0 |
| ABO | 3.24 | 1.21E-03 | 2.97E-02 | 1.07 | 1.03 | 1.11 | 8.36E-04 | 190 | 1.98E-02 | 8.18E-01 |  |  |
| MAN2B2 | -3.22 | 1.27E-03 | 3.01E-02 | 0.93 | 0.90 | 0.97 | 2.95E-04 | 259 | 1.46E-02 | 1.97E-01 |  |  |
| FLRT3 | -3.23 | 1.25E-03 | 3.01E-02 | 0.96 | 0.93 | 0.98 | 2.07E-03 | 351 | 4.31E-01 | 6.68E-01 | 0.07 | 0.29 |
| TNFRSF1A | 3.20 | 1.37E-03 | 3.13E-02 | 1.24 | 1.09 | 1.41 | 1.32E-03 | 130 |  |  | 0.28 | 0.37 |
| INHBC | -3.20 | 1.36E-03 | 3.13E-02 | 0.66 | 0.51 | 0.85 | 1.38E-03 | 32 |  |  | 0.2 | 0.43 |
| LY75 | -3.18 | 1.50E-03 | 3.32E-02 | 0.96 | 0.92 | 1.01 | 9.99E-02 | 219 | 8.43E-01 | 6.06E-01 |  |  |
| STX7 | -3.13 | 1.76E-03 | 3.83E-02 | 0.87 | 0.78 | 0.96 | 7.87E-03 | 101 |  |  | 0.1 | 0.04 |
| ITLN1 | -3.08 | 2.10E-03 | 4.49E-02 | 0.81 | 0.62 | 1.05 | 1.09E-01 | 34 |  |  |  |  |
| ENTPD6 | 3.06 | 2.21E-03 | 4.65E-02 | 1.17 | 1.05 | 1.30 | 4.18E-03 | 89 |  |  | 0.03 | 0.58 |
| MTAP | 3.04 | 2.34E-03 | 4.77E-02 | 1.58 | 1.18 | 2.12 | 2.38E-03 | 26 |  |  | 0.13 | 0.45 |
| **UC** |  |  |  |  |  |  |  |  |  |  |  |  |
| HLA-DQA2 | 7.38 | 1.58E-13 | 2.10E-10 | 1.01 | 0.87 | 1.17 | 9.11E-01 | 261 | 1.54E-01 | 5.65E-02 |  |  |
| MST1 | -6.97 | 3.07E-12 | 2.04E-09 | 0.85 | 0.79 | 0.90 | 6.22E-08 | 278 | 7.04E-01 | 1.62E-02 | 0.29 | 0.71 |
| IL23R | 6.81 | 9.61E-12 | 4.25E-09 | 1.01 | 0.75 | 1.37 | 9.34E-01 | 38 |  |  |  |  |
| FCGR3B | 6.47 | 9.52E-11 | 3.16E-08 | 1.20 | 0.96 | 1.50 | 1.15E-01 | 37 | 6.38E-03 | 3.17E-02 |  |  |
| STAT3 | -6.07 | 1.27E-09 | 3.37E-07 | 0.78 | 0.50 | 1.20 | 2.54E-01 | 339 |  |  |  |  |
| FCGR3A | 5.91 | 3.45E-09 | 6.54E-07 | 1.04 | 0.93 | 1.16 | 5.16E-01 | 255 | 3.85E-01 | 5.06E-01 |  |  |
| AIF1 | -5.92 | 3.23E-09 | 6.54E-07 | 0.78 | 0.66 | 0.92 | 2.33E-03 | 56 | 2.35E-04 | 6.16E-06 | 1 | 0 |
| FCGR2A | 5.66 | 1.47E-08 | 2.44E-06 | 0.96 | 0.85 | 1.08 | 4.96E-01 | 257 | 1.33E-01 | 1.83E-01 |  |  |
| AGER | 4.94 | 7.93E-07 | 1.17E-04 | 1.47 | 0.95 | 2.27 | 8.47E-02 | 200 |  |  |  |  |
| MAPKAPK2 | 4.50 | 6.89E-06 | 9.14E-04 | 0.85 | 0.63 | 1.15 | 2.89E-01 | 137 |  |  |  |  |
| VSIR | -4.30 | 1.71E-05 | 2.06E-03 | 0.89 | 0.83 | 0.95 | 2.77E-04 | 163 | 1.18E-02 | 3.64E-01 | 0.03 | 0.88 |
| IL1R2 | 4.25 | 2.12E-05 | 2.16E-03 | 1.14 | 1.06 | 1.22 | 1.63E-04 | 256 | 3.17E-02 | 7.34E-01 |  |  |
| HGFAC | -4.26 | 2.06E-05 | 2.16E-03 | 0.93 | 0.88 | 0.98 | 1.12E-02 | 440 | 4.65E-03 | 1.89E-01 | 0.01 | 0.98 |
| PCOLCE2 | 4.13 | 3.59E-05 | 3.40E-03 | 1.05 | 0.98 | 1.12 | 2.01E-01 | 140 | 1.68E-02 | 5.05E-02 |  |  |
| PRKCB | 4.04 | 5.33E-05 | 4.71E-03 | 1.19 | 1.08 | 1.31 | 6.20E-04 | 126 | 2.61E-01 | 5.26E-01 | 0.96 | 0.04 |
| MICB | -4.02 | 5.87E-05 | 4.86E-03 | 0.95 | 0.88 | 1.03 | 2.18E-01 | 494 | 4.69E-01 | 6.23E-02 |  |  |
| PIGR | -3.94 | 8.13E-05 | 6.34E-03 | 0.78 | 0.67 | 0.90 | 7.14E-04 | 88 |  |  | 1 | 0 |
| PARK7 | -3.74 | 1.86E-04 | 1.30E-02 | 0.86 | 0.75 | 0.98 | 2.86E-02 | 194 | 2.74E-02 | 1.72E-01 |  |  |
| CADM2 | -3.62 | 2.90E-04 | 1.92E-02 | 0.64 | 0.51 | 0.80 | 1.46E-04 | 63 |  |  | 0.05 | 0.89 |
| FOLR2 | -3.55 | 3.85E-04 | 2.43E-02 | 0.80 | 0.66 | 0.96 | 1.79E-02 | 34 | 9.98E-03 | 3.78E-02 | 0.06 | 0.62 |
| CTSZ | -3.40 | 6.74E-04 | 4.06E-02 | 1.13 | 0.87 | 1.47 | 3.49E-01 | 71 |  |  |  |  |
| **CD** |  |  |  |  |  |  |  |  |  |  |  |  |
| IL23R | 12.69 | 6.84E-37 | 9.07E-34 |  |  |  |  |  |  |  |  |  |
| MST1 | -9.21 | 3.41E-20 | 2.26E-17 | 0.83 | 0.77 | 0.90 | 6.08E-06 | 278 | 9.50E-01 | 5.24E-02 | 0.13 | 0.87 |
| STAT3 | -6.47 | 1.01E-10 | 4.46E-08 | 1.25 | 0.80 | 1.95 | 3.20E-01 | 339 |  |  |  |  |
| C2 | -6.21 | 5.39E-10 | 1.43E-07 | 0.96 | 0.71 | 1.29 | 7.67E-01 | 100 | 2.78E-02 | 1.46E-02 |  |  |
| MICB | 6.22 | 5.05E-10 | 1.43E-07 | 0.89 | 0.72 | 1.11 | 2.95E-01 | 494 | 9.51E-01 | 6.57E-01 |  |  |
| IL18R1 | -6.15 | 7.57E-10 | 1.67E-07 | 1.01 | 0.94 | 1.08 | 8.37E-01 | 465 | 7.75E-01 | 8.30E-01 |  |  |
| HINT1 | -5.85 | 4.89E-09 | 9.26E-07 |  |  |  |  |  |  |  |  |  |
| PLAU | 5.65 | 1.59E-08 | 2.64E-06 | 0.89 | 0.66 | 1.19 | 4.26E-01 | 131 | 5.06E-01 | 2.72E-01 |  |  |
| FLRT3 | -5.59 | 2.32E-08 | 3.42E-06 | 0.90 | 0.87 | 0.93 | 4.99E-08 | 351 | 1.39E-03 | 2.29E-01 | 0.03 | 0.97 |
| RIPK2 | -5.12 | 2.99E-07 | 3.96E-05 |  |  |  |  |  |  |  |  |  |
| C7 | 4.39 | 1.13E-05 | 1.36E-03 | 1.14 | 1.06 | 1.23 | 8.41E-04 | 203 | 7.01E-02 | 8.76E-01 | 1 | 0 |
| IL1RL1 | 4.29 | 1.77E-05 | 1.96E-03 | 1.03 | 0.90 | 1.18 | 6.60E-01 | 271 | 4.34E-01 | 2.91E-01 |  |  |
| HGFAC | -4.27 | 1.96E-05 | 2.00E-03 | 0.94 | 0.90 | 0.97 | 9.72E-04 | 440 | 1.21E-02 | 7.85E-01 | 0.45 | 0.21 |
| TNFSF15 | -4.21 | 2.54E-05 | 2.41E-03 | 0.77 | 0.57 | 1.05 | 1.03E-01 | 45 |  |  |  |  |
| TNFSF8 | 4.11 | 4.03E-05 | 3.56E-03 | 1.08 | 0.95 | 1.21 | 2.35E-01 | 125 |  |  |  |  |
| IRF3 | -3.96 | 7.63E-05 | 6.32E-03 | 0.90 | 0.84 | 0.97 | 5.92E-03 | 181 | 1.02E-01 | 4.47E-01 | 0.85 | 0.05 |
| IL12B | -3.94 | 8.24E-05 | 6.43E-03 | 1.03 | 0.81 | 1.30 | 8.15E-01 | 213 | 1.12E-01 | 9.14E-02 |  |  |
| HSPA1A | -3.80 | 1.45E-04 | 1.07E-02 |  |  |  |  |  |  |  |  |  |
| LRRC32 | 3.72 | 1.99E-04 | 1.39E-02 | 1.12 | 0.86 | 1.46 | 3.90E-01 | 26 |  |  |  |  |
| APOM | -3.61 | 3.03E-04 | 2.01E-02 | 0.88 | 0.74 | 1.04 | 1.36E-01 | 120 | 1.82E-01 | 6.00E-01 |  |  |
| ABO | 3.59 | 3.26E-04 | 2.06E-02 | 1.11 | 1.06 | 1.16 | 3.96E-05 | 190 | 1.08E-03 | 5.29E-01 | 0.22 | 0.46 |
| PPIH | -3.56 | 3.72E-04 | 2.24E-02 | 0.72 | 0.54 | 0.96 | 2.60E-02 | 45 |  |  |  |  |
| CFB | 3.50 | 4.69E-04 | 2.70E-02 | 1.07 | 0.97 | 1.17 | 1.67E-01 | 246 | 1.83E-02 | 7.55E-02 |  |  |
| PRKCB | 3.49 | 4.89E-04 | 2.70E-02 | 1.16 | 1.06 | 1.29 | 2.39E-03 | 126 | 3.53E-03 | 4.72E-02 | 0.12 | 0.14 |
| ADK | -3.45 | 5.55E-04 | 2.83E-02 | 0.84 | 0.61 | 1.16 | 2.85E-01 | 35 |  |  |  |  |
| TNFRSF1A | 3.46 | 5.44E-04 | 2.83E-02 | 1.35 | 1.14 | 1.60 | 5.32E-04 | 130 |  |  | 1 | 0 |
| C9 | 3.43 | 6.09E-04 | 2.99E-02 | 1.07 | 0.90 | 1.27 | 4.64E-01 | 82 | 3.18E-01 | 4.20E-01 |  |  |
| GKN2 | 3.37 | 7.41E-04 | 3.51E-02 | 1.11 | 1.01 | 1.22 | 3.45E-02 | 241 | 5.83E-04 | 5.47E-02 |  |  |
| CXCL10 | -3.35 | 8.20E-04 | 3.75E-02 | 0.62 | 0.42 | 0.91 | 1.34E-02 | 26 |  |  | 0.11 | 0.34 |
| SERPINF2 | -3.27 | 1.09E-03 | 4.82E-02 | 0.87 | 0.77 | 0.98 | 1.93E-02 | 274 |  |  |  |  |

**Supplementary Table S3. Detailed data on MR analysis results of IBD**

| protein | method | beta | se | OR | (95%CI) | | num_SNP | pval | FDR |
| --- | --- | --- | --- | --- | --- | --- | --- | --- | --- |
| MST1 | IVW | -0.19 | 0.04 | 0.82 | 0.77 | 0.88 | 17 | 6.14E-08 | 3.62E-06 |
| PARK7 | IVW | -0.21 | 0.04 | 0.81 | 0.75 | 0.89 | 10 | 1.76E-06 | 5.18E-05 |
| NADK | Wald Ratio | -0.17 | 0.04 | 0.84 | 0.78 | 0.91 | 1 | 3.25E-05 | 6.40E-04 |
| RIPK2 | Wald Ratio | -0.48 | 0.12 | 0.62 | 0.49 | 0.78 | 1 | 6.22E-05 | 9.17E-04 |
| TALDO1 | Wald Ratio | -0.51 | 0.13 | 0.60 | 0.46 | 0.78 | 1 | 1.14E-04 | 1.35E-03 |
| AIF1 | IVW | -0.24 | 0.06 | 0.79 | 0.70 | 0.89 | 3 | 2.02E-04 | 1.47E-03 |
| IRF3 | IVW | -0.09 | 0.02 | 0.92 | 0.88 | 0.96 | 7 | 2.04E-04 | 1.47E-03 |
| EPHB4 | Wald Ratio | 0.18 | 0.05 | 1.20 | 1.09 | 1.32 | 1 | 2.24E-04 | 1.47E-03 |
| CADM2 | Wald Ratio | -0.34 | 0.09 | 0.71 | 0.60 | 0.86 | 1 | 2.77E-04 | 1.58E-03 |
| RHOC | IVW | 0.19 | 0.05 | 1.21 | 1.09 | 1.34 | 2 | 3.39E-04 | 1.67E-03 |
| PLG | IVW | 0.09 | 0.02 | 1.09 | 1.04 | 1.15 | 7 | 4.76E-04 | 2.16E-03 |
| AGER | IVW | 0.13 | 0.04 | 1.14 | 1.06 | 1.23 | 2 | 6.53E-04 | 2.75E-03 |
| MMP9 | IVW | -0.14 | 0.04 | 0.87 | 0.80 | 0.94 | 2 | 9.70E-04 | 3.55E-03 |
| ICAM5 | IVW | 0.06 | 0.02 | 1.06 | 1.03 | 1.10 | 9 | 1.02E-03 | 3.55E-03 |
| MXRA8 | Wald Ratio | -0.33 | 0.10 | 0.72 | 0.59 | 0.88 | 1 | 1.24E-03 | 4.06E-03 |
| TNFRSF1A | Wald Ratio | 0.21 | 0.07 | 1.24 | 1.09 | 1.41 | 1 | 1.32E-03 | 4.07E-03 |
| INHBC | Wald Ratio | -0.42 | 0.13 | 0.66 | 0.51 | 0.85 | 1 | 1.38E-03 | 4.07E-03 |
| FLRT3 | IVW | -0.05 | 0.01 | 0.96 | 0.93 | 0.98 | 12 | 2.07E-03 | 5.55E-03 |
| BPI | IVW | 0.06 | 0.02 | 1.06 | 1.02 | 1.10 | 7 | 2.18E-03 | 5.60E-03 |
| TYMP | IVW | -0.12 | 0.04 | 0.89 | 0.83 | 0.96 | 3 | 2.33E-03 | 5.62E-03 |
| MTAP | Wald Ratio | 0.46 | 0.15 | 1.58 | 1.18 | 2.12 | 1 | 2.38E-03 | 5.62E-03 |
| FCGR2B | IVW | -0.11 | 0.04 | 0.90 | 0.84 | 0.96 | 15 | 2.63E-03 | 5.96E-03 |
| FCGR2A | IVW | -0.11 | 0.04 | 0.89 | 0.83 | 0.96 | 13 | 3.33E-03 | 7.27E-03 |
| ENTPD6 | IVW | 0.16 | 0.06 | 1.17 | 1.05 | 1.30 | 2 | 4.18E-03 | 8.80E-03 |
| GUCA2B | Wald Ratio | 0.22 | 0.08 | 1.25 | 1.07 | 1.46 | 1 | 5.85E-03 | 1.19E-02 |
| PLCG2 | IVW | 0.11 | 0.04 | 1.12 | 1.03 | 1.21 | 3 | 6.45E-03 | 1.23E-02 |
| PLAU | IVW | -0.13 | 0.05 | 0.88 | 0.81 | 0.97 | 5 | 7.50E-03 | 1.38E-02 |
| STX7 | IVW | -0.14 | 0.05 | 0.87 | 0.78 | 0.96 | 2 | 7.87E-03 | 1.41E-02 |
| IL1R2 | IVW | 0.18 | 0.07 | 1.20 | 1.04 | 1.38 | 4 | 1.35E-02 | 2.28E-02 |
| PIGR | IVW | -0.14 | 0.06 | 0.87 | 0.78 | 0.98 | 2 | 1.75E-02 | 2.86E-02 |
| ERAP2 | IVW | 0.07 | 0.03 | 1.07 | 1.01 | 1.14 | 16 | 2.26E-02 | 3.61E-02 |
| LY9 | IVW | 0.09 | 0.04 | 1.09 | 1.01 | 1.18 | 7 | 3.07E-02 | 4.77E-02 |

**Supplementary Table S4. Detailed data on MR analysis results of UC**

| protein | method | beta | se | OR | (95%CI) | | num_SNP | pval | FDR |
| --- | --- | --- | --- | --- | --- | --- | --- | --- | --- |
| MST1 | IVW | -0.17 | 0.03 | 0.84 | 0.79 | 0.90 | 17 | 6.22E-08 | 1.31E-06 |
| CADM2 | Wald Ratio | -0.45 | 0.12 | 0.64 | 0.51 | 0.80 | 1 | 1.46E-04 | 1.14E-03 |
| VSIR | IVW | -0.12 | 0.03 | 0.89 | 0.83 | 0.95 | 6 | 2.77E-04 | 1.46E-03 |
| PRKCB | IVW | 0.17 | 0.05 | 1.19 | 1.08 | 1.31 | 3 | 6.20E-04 | 2.50E-03 |
| PIGR | IVW | -0.25 | 0.07 | 0.78 | 0.67 | 0.90 | 2 | 7.14E-04 | 2.50E-03 |
| AIF1 | IVW | -0.25 | 0.08 | 0.78 | 0.66 | 0.92 | 3 | 2.33E-03 | 7.00E-03 |
| HGFAC | IVW | -0.07 | 0.03 | 0.93 | 0.88 | 0.98 | 11 | 1.12E-02 | 2.93E-02 |
| FOLR2 | IVW | -0.23 | 0.10 | 0.80 | 0.66 | 0.96 | 3 | 1.79E-02 | 4.18E-02 |

**Supplementary Table S5. Detailed data on MR analysis results of CD**

| protein | method | beta | se | OR | (95%CI) | | num_SNP | pval | FDR |
| --- | --- | --- | --- | --- | --- | --- | --- | --- | --- |
| FLRT3 | IVW | -0.11 | 0.02 | 0.90 | 0.87 | 0.93 | 12 | 4.99E-08 | 1.30E-06 |
| MST1 | IVW | -0.19 | 0.04 | 0.83 | 0.77 | 0.90 | 17 | 6.08E-06 | 7.90E-05 |
| ABO | IVW | 0.10 | 0.02 | 1.11 | 1.06 | 1.16 | 10 | 3.96E-05 | 3.43E-04 |
| TNFRSF1A | Wald Ratio | 0.30 | 0.09 | 1.35 | 1.14 | 1.60 | 1 | 5.32E-04 | 3.46E-03 |
| C7 | IVW | 0.13 | 0.04 | 1.14 | 1.06 | 1.23 | 10 | 8.41E-04 | 4.21E-03 |
| HGFAC | IVW | -0.06 | 0.02 | 0.94 | 0.90 | 0.97 | 11 | 9.72E-04 | 4.21E-03 |
| PRKCB | IVW | 0.15 | 0.05 | 1.16 | 1.06 | 1.29 | 3 | 2.39E-03 | 8.88E-03 |
| IRF3 | IVW | -0.10 | 0.04 | 0.90 | 0.84 | 0.97 | 7 | 5.92E-03 | 1.92E-02 |
| CXCL10 | Wald Ratio | -0.48 | 0.19 | 0.62 | 0.42 | 0.91 | 1 | 1.34E-02 | 3.86E-02 |
